# Isolation and Characterization of Human Gut Bacteria Capable of Extracellular Electron Transport by Electrochemical Techniques

**DOI:** 10.3389/fmicb.2018.03267

**Published:** 2019-01-15

**Authors:** Divya Naradasu, Waheed Miran, Mitsuo Sakamoto, Akihiro Okamoto

**Affiliations:** ^1^International Center for Materials Nanoarchitectonics (WPI-MANA), National Institute for Materials Science, Tsukuba, Japan; ^2^Department of Advanced Interdisciplinary Studies, Research Center for Advanced Science and Technology, Graduate School of Engineering, The University of Tokyo, Tokyo, Japan; ^3^Microbe Division/Japan Collection of Microorganisms, RIKEN BioResource Research Center, Tsukuba, Japan; ^4^PRIME, Japan Agency for Medical Research and Development (AMED), Tsukuba, Japan; ^5^Center for Sensor and Actuator Material, National Institute for Materials Science (NIMS), Tsukuba, Japan

**Keywords:** gut microbes, electromicrobiology, fermentative bacteria, electrochemical enrichment, extracellular electron transfer

## Abstract

Microorganisms are known to exhibit extracellular electron transfer (EET) in a wide variety of habitats. However, as for the human microbiome which significantly impacts our health, the role and importance of EET has not been widely investigated. In this study, we enriched and isolated the EET-capable bacteria from human gut microbes using an electrochemical enrichment method and examined whether the isolates couple EET with anaerobic respiration or fermentation. Upon the use of energy-rich or minimum media (with acetate or lactate) for electrochemical enrichment with the human gut sample at an electrode potential of +0.4 V [vs. the standard hydrogen electrode (SHE)], both culture conditions showed significant current production. However, EET-capable pure strains were enriched specifically with minimum media, and subsequent incubation using the δ-MnO_2_-agar plate with lactate or acetate led to the isolation of two EET-capable microbial strains, *Gut-S1* and *Gut-S2*, having 99% of 16S rRNA gene sequence identity with *Enterococcus avium* (*E. avium*) and *Klebsiella pneumoniae* (*K. pneumoniae*), respectively. While the enrichment involved anaerobic respiration with acetate and lactate, further electrochemistry with *E. avium* and *K. pneumoniae* revealed that the glucose fermentation was also coupled with EET. These results indicate that EET couples not only with anaerobic respiration as found in environmental bacteria, but also with fermentation in the human gut.

## Introduction

Extracellular electron transfer (EET) mechanisms have evolved in microorganisms as an anaerobic metabolic strategy that can be coupled to the reduction of extracellular solid materials (Myers and Nealson, 1988; Shi et al., 2016). EET mechanisms are proposed to be mediated by cell surface transmembrane cytochromes, exogenous or endogenous soluble redox-active compounds, or electrically conductive nanowires (Shi et al., 2016). The significance of EET during anaerobic respiration has provided a reasonable explanation for not only microbial energy conservation and physiology, but also for their interaction with the environment. While EET has been well characterized in terms of its mechanistic basis, mainly in two model bacterial strains, *Geobacter sulfurreducens* PCA and *Shewanella oneidensis* MR-1 (Lovley and Phillips, 1988; Myers and Nealson, 1988), electrochemical enrichment studies, combined with 16S rRNA-based assessments in a variety of environments, advocate that more physiologically and phylogenetically diverse microorganisms may be capable of using exterior surfaces as electron acceptors. However, it is vital to mention that the survival or enrichment of a microbe on an electrode is not the ultimate evidence for their EET ability, and hence, further electrochemical characterization is essential to probe the EET processes after the isolation of the microbes. A number of enrichments have resulted in the isolation of pure cultures that can accomplish EET with electrodes, indicating that EET may be advantageous in a wide variety of habitats (Zuo et al., 2008; Fedorovich et al., 2009; Logan, 2009; Rowe et al., 2017). Moreover, bacteria that utilize fermentation as their main metabolic pathway have also been isolated and characterized for their EET capabilities (Khan et al., 2012; Zhou et al., 2016; Kumar P. et al., 2018).

In anaerobic environments with substantially reductive condition such as the human gut (Edwards et al., 1985), fermentation is the primary mechanism of microbial metabolism, in which the redox cycling of biological electron carriers, such as nicotinamide adenine dinucleotide (NADH), drives the intracellular oxidation and reduction of organic substrates. As fermentation does not require extracellular electron acceptors for the termination of metabolism, the energy gain under such conditions is potentially lower than that of respiratory metabolism; therefore, the possibility for EET to increase the rate of NAD^+^ regeneration and fermentative metabolism may be important for these microbes to increase their net energy gain and compete with other respiratory bacteria (Okamoto et al., 2017). In fact, a few studies have shown that fermentative gut microbes are capable of EET using soluble electron carrier molecules (Khan et al., 2012; Keogh et al., 2018; Light et al., 2018; Pankratova et al., 2018). However, by simply studying isolated bacterial cultures, it is impossible to examine which bacteria primarily rely on EET-coupled metabolism in the human gut and to study the ecophysiological importance of EET coupled with fermentation, compared to anaerobic respiration, which is also abundant in the gut environment (Rey et al., 2013). Here, we examined the growth competition between fermentative and respiratory bacteria on an electrode surface that enriches for EET-capable bacteria. Specifically, we performed electrochemical enrichment, which was initiated using a diluted gut microbial community, by employing two different medium conditions that biased for either fermentation or anaerobic respiration. The isolated bacterial strains were characterized by electrochemical assays for their metabolism associated with current production and the EET mechanism.

## Materials and Methods

### Electrochemical Cell Operation and Medium Composition

Electrochemical measurements were performed in single-chamber and three-electrode reactors. Tin-doped In_2_O_3_ (ITO) grown on a glass substrate by spray pyrolysis deposition was used as the working electrode (WE) having a surface area of 3.1 cm^2^, and thickness 1.1 mm. The WEs were placed at the bottom of the reactor with sealing gaskets to avoid any leakage. A platinum wire (approximate diameter of 0.1 mm) and Ag/AgCl (sat. KCl) were used as counter and reference electrodes, respectively. Electrochemical experiments were conducted in a COY anaerobic chamber filled with 100% N_2_. Electrochemical analysis techniques such as single-potential amperometry (SA) and differential pulse voltammetry (DPV) were measured with an automatic polarization system (VMP3, Bio-Logic Science Instruments). DPV was measured under the following conditions: pulse increment, 5.0 mV; pulse amplitude, 50 mV; pulse width, 300 ms; and pulse period, 5.0 s. The electrochemical cell was maintained at 37°C throughout the experiment and the WE was poised at +0.2 V [vs. Ag/AgCl (sat. KCl)] reference electrode for SA.

Gifu Anaerobic Medium (GAM Broth), which is known for providing reducing conditions and adequate anaerobiosis, was used as an energy rich medium for the enrichment of gut microbes. Defined medium 1 (DM1), used as a minimum medium for EET strains enrichment and initial electrochemical characterization experiments had the following composition (L^-1^): NH_4_Cl: 1 g; MgCl_2_ 6H_2_O: 0.8 g; CaCl_2_.2H_2_O: 0.1 g; KH_2_PO_4_: 0.5 g; yeast extract: 1 g; NaHCO_3_: 1 g; trace mineral mix: 10 mL [with the following composition (L^-1^): Nitrilotriacetic acid: 1.5 g, MgSO_4_ 7H_2_O: 3 g, MnSO_4_ H_2_O: 0.5 g, NaCl: 1 g, FeSO4 7H_2_O: 0.1 g, CoSO_4_ 7H_2_O: 0.18 g, CaCl_2_ 2H_2_O: 0.1 g, ZnSO_4_ 7H_2_O: 0.18 g, CuSO_4_ 5H_2_O: 0.01 g, KAl (SO_4_)_2_ 12H_3_BO_3_: 0.02 g, Na_2_MoO_4_ 2H_2_O: 0.01 g, NiCl_2_ 6H_2_O: 0.03 g, Na_2_SeO^3^ 5H_2_O: 0.3 mg, Na_2_WO_4_ 2H_2_O: 0.4 mg; first dissolved the nitrilotriacetic acid and adjusted the pH to 6.5 with KOH, then added the minerals. Finally, pH was adjusted to 7.0 with KOH] and trace vitamin mix: 10 mL [with the following composition (L^-1^): Biotin: 2 mg, Folic acid: 2 mg, Pyridoxine-HCl: 10 mg, Thiamine-HCl: 5 mg, Riboflavin: 5 mg, Nicotinic acid: 5 mg, D-Ca-pantothenate: 5 mg, Vitamin B12: 0.1 mg, *p*-Aminobenzoic acid: 5 mg, Lipoic acid: 5 mg]. Acetate (30 mM) or lactate (30 mM) was used as electron donors. DM1 exclusive of NaHCO_3_, trace mineral, and trace vitamin was autoclaved first, and pH was adjusted to 7.2 after adding the remaining components. The final medium was filtered using 0.22-μm-pore-size filters and deaerated by purging it with 100% N_2_ for 15 min prior to use for experiments. A fine powder of δ-MnO_2_ was synthesized as previously reported (Burdige and Nealson, 1985). Briefly, Mn^2+^ was oxidized by permanganate under basic conditions, and the product was washed and resuspended in distilled water. The solid was freeze-dried for storage, and resuspended in sterilized solution before use in agar plate.

Defined minimum medium 2 (DM2), used as an electrolyte for EET experiments had the following composition (L^-1^): NH_4_Cl: 1 g; MgCl_2_.6H_2_O: 0.2 g; CaCl_2_.2H_2_O: 0.08 g; yeast extract: 0.5 g; NaHCO_3_: 2.5 g; NaCl: 10 g; and HEPES buffer: 7.2 g. Acetate (10 mM) or lactate (10 mM) was used as electron donors for EET experiments with DM2. This medium was also autoclaved and deaerated prior to the electrochemical experiments. A total of 5 mL of medium (including strain culture) was used in the electrochemical reactor for all experiments. During the electrochemical measurements, the reactor was operated at 37°C throughout the experiment without any agitation.

### Electrochemical Enrichment of Human Gut Sample

This study was approved by the RIKEN Ethics Committee. A fecal sample was obtained from a healthy volunteer (45–50 years old). A written informed consent agreement signed by the volunteer was obtained before the experiment. A 0.5 g of fecal sample was suspended in 4.5 mL of pre-reduced phosphate buffer saline (PBS) and then serially diluted in 10-fold steps. The sample was diluted to a concentration of 10^-7^ (v/v) and 0.1 mL of this diluted microbial consortium was then added to the electrochemical reactor with 4.9 mL of GAM medium or DM1 in which the electrolyte temperature was maintained at 37°C, and the WE was poised at +0.2 V throughout the enrichment.

At the end of electrochemical enrichment with GAM, or DM1 having acetate or lactate, the WE surface was washed twice with PBS, and subsequently the electrode-attached biomass from each WE was streaked on separate agar plates which were made with GAM or DM1 having acetate and lactate, and contained 50–60 mM δ-MnO_2_. The plates were incubated at 37°C under an H_2_/CO_2_/N_2_ (1:1:8 v/v) gas mixture. Given the microbial reduction of δ-MnO_2_ generate transparent spot in the dark brown agar plate, this visual clue was used to identify the colony of EET-capable bacteria. The schematics of isolation procedure are shown in Supplementary Figure S1. After 2–4 days of incubation, the colonies that formed the transparent spots were sub-cultured on an Eggerth Gagnon agar (Merck) supplemented with 5% horse blood at 37°C under the same conditions as mentioned above, and single colonies were picked up for further analysis by sequencing. The strains isolated from agar plates having acetate and lactate were named as *Gut-S1* and *Gut-S2*, respectively.

### Cell Cultures Harvesting

The isolated strains *Gut-S1* and *Gut-S2* were pre-cultivated in 40 mL of Lysogeny broth (LB) in butyl-rubber-stoppered vials at 37°C with an anoxic headspace of CO_2_/N_2_ (20:80 v/v). Microbial cultures were harvested in the late exponential phase when the OD_600_ was about 1.0. The cultures were centrifuged at 7800 rpm at 37°C for 10 min in a 50 mL falcon tubes. The resultant cell pellet was washed twice with defined media (DM1 or DM2) by resuspending and centrifugation. The resuspended cells in defined media were then added into the reactors to a final OD_600_ of 0.1.

### Metabolites Determination

Samples for metabolites were collected from the electrochemical cells at every 8-h time interval for 24-h during the electrochemical operations. The collected samples were filtered using 0.22-μm-pore-size filters to remove the cells and stored at -20°C until further analysis. For metabolite analysis, samples were diluted 100 times with distilled water. Metabolic products were quantified by using an ion chromatography (IC) system (HIC-20Asuper, Shimadzu Corporation, Japan). Fifty microliter samples were injected, and the anions were analyzed in non-suppressor mode. Shim-pack IC-A3 and Shim-pack IC-GA3 (Shimadzu Corporation, Japan) were used as the analytical column and guard column, respectively. The mobile phase contained 8 mM *p*-hydroxybenzoic acid, 3.2 mM Bis–Tris, and 50 mM boric acid, and the flow rate was 1.2 mL/min. The column temperature was maintained at 40°C, and the detector (CDD-10A_SP_) parameters were set according to the manufacturer’s guidelines. Peak area analysis was conducted by Shimadzu analytical workstation software LabSolutions provided by the manufacturer. The standard curves showed sufficiently high linearity (*R*^2^ = 0.999). Glucose concentrations were measured by using a glucose assay kit (GAGO-20, Sigma–Aldrich) according to the manufacturer’s protocol.

### DNA Isolation and Phylogenetic Tree

The discrete colonies that were sub-cultured from the transparent spots of δ-MnO_2_ were analyzed by 16S rRNA gene sequencing. The forward and reverse primers for PCR were 27 F (5′-AGA GTT TGA TCC TGG CTC AG-3′) and 1492 R (5′-GGT TAC CTT GTT ACG ACT T-3′). The 16S rRNA gene sequences were compared with the sequences of closely related strains by using the BLAST program in the GenBank database. These sequence data were deposited in NCBI GenBank under accession numbers MK051424 and MK051423 for *Gut-S1* and *Gut-S2*, respectively. For the construction of a 16S rRNA phylogenetic tree, 16S rRNA sequences were collected from the NCBI nucleotide database, aligned using MUSCLE (Edgar, 2004), and analyzed by the neighbor-joining method (Saitou and Nei, 1987) using Molecular Evolutionary Genetics Analysis package (MEGA, version X.0) (Kumar S. et al., 2018).

### Scanning Electron Microscopy

For the scanning electron microscopy, ITO electrodes were removed from the reactors after performing the electrochemical measurements. Microbial fixation on electrodes was carried out with 2.5% glutaraldehyde for 10 min in the dark at room temperature. This was followed by washing three times in 0.1 M phosphate buffer (pH 7.4) for 15 min each. These washed samples were then dehydrated in 30, 50, 70, 90, and 100% ethanol gradients (prepared in the 0.1 M buffer) for 15 min each. Ethanol gradient dehydrated samples were exchanged thrice with 100% t-butanol and finally freeze-dried under vacuum. The dried samples were coated with platinum and then observed using a Keyence VE-9800 microscope.

### Supernatant Exchange During Current Producing Condition

The electron transfer mechanism of gut microbes was evaluated using a medium-replacement experiment used to test electron transfer in environmental model microbes *S. oneidensis* MR1 (Marsili et al., 2008). Here, the medium in the electrochemical cell was removed; the biofilm was rinsed with N_2_-sparged DM twice at each replacement; and the headspace was continuously sparged with N_2_ during each replacement to avoid the leakage of oxygen into the electrochemical cell. The cell was refilled with N_2_-sparged sterile medium (10 mM acetate or lactate). DPV measurements were performed as previously described to detect the redox molecules (Okamoto et al., 2009) before and after supernatant exchanges.

## Results

### Electrochemical Enrichment and Isolation of EET-Capable Human Gut Microbes

For the isolation of fermentative bacteria capable of EET, we initiated electrochemical enrichment in GAM medium with a microbial consortium sample collected from the human gut and diluted it to a concentration of 10^-7^ (v/v) at 37°C. Current production (*I*_c_) was measured to be approximately 0.3 – 0.4 μA cm^-2^ for each cycle at around 48 h, and it gradually decreased. After 1 week of incubation, we replaced the spent medium with fresh GAM medium, and after another week of electrochemical incubation, the electrode surface was washed to collect the enriched bacterial cells. Although the subsequent plating resulted in the formation of many colonies, none of the colonies showed transparent spots on the black δ-MnO_2_ agar plates, indicating that MnO_2_ was not reduced, and hence, no colonies of EET-capable bacteria were isolated. This unsuccessful result was probably due to the fact that the GAM medium grew too many fermentative bacterial cells that were incapable of EET in bulk, and the EET-capable bacteria that were potentially enriched on the electrode became a minority.

**FIGURE 1:**
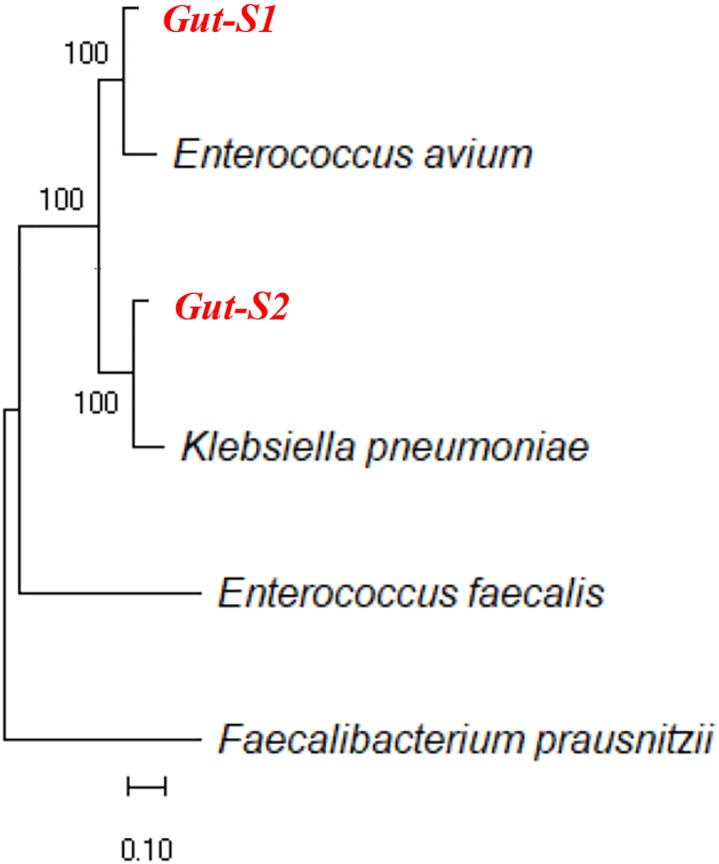
Ribosomal RNA gene sequences of isolated electrogenic microbes (isolated in bold red) were aligned with the representative gut microbial sequences previously reported for their EET capability. Alignment was carried out using MUSCLE and the neighbor-joining method was employed for phylogenetic tree construction.

Next, we used DM1, containing either 30 mM acetate or 30 mM lactate as an electron donor, to enrich for bacteria that can couple EET with anaerobic respiration during the same time frame as the enrichment with the GAM medium. Although the *I*_c_ only reached 15–20 nA cm^-2^ during the DM1 cycles (Supplementary Figures S2a,b), transparent spots were observed on the δ-MnO_2_ agar plates, after the incubation of the cells collected from the reactors enriched with either lactate or acetate. All of the colonies with the transparent spots looked identical, and one colony from each plate was analyzed for its 16S rRNA gene sequence. Sequence alignment using NCBI showed that the strain isolated from the acetate-fed reactor belongs to the genus *Enterococcus*, and the strain isolated from the lactate-fed reactor belongs to the genus *Klebsiella*. Analysis of the 16S rRNA gene sequences of the strain *Gut-S1* using the GenBank database showed that it has more than 99% identity with *Enterococcus avium*, and analysis of the 16S rRNA gene sequences of the lactate-enriched strain *Gut-S2* showed that it has more than 99% identity with *Klebsiella pneumoniae*. The ribosomal RNA gene sequences of these isolated strains were aligned with representative microbial community sequences from human gut microbes (*Enterococcus faecalis* and *Faecalibacterium prausnitzii*) that had been previously reported to have EET capability. The phylogenetic analysis is shown in Figure 1. *E. avium* and *K. pneumoniae*, which are similar to *Gut-S1* and *Gut-S2*, are Gram-positive and Gram-negative strains, respectively, and both are fermentative under anaerobic conditions.

### Electrochemical Characterization of the Metabolism in the Isolated Strains

**FIGURE 2:**
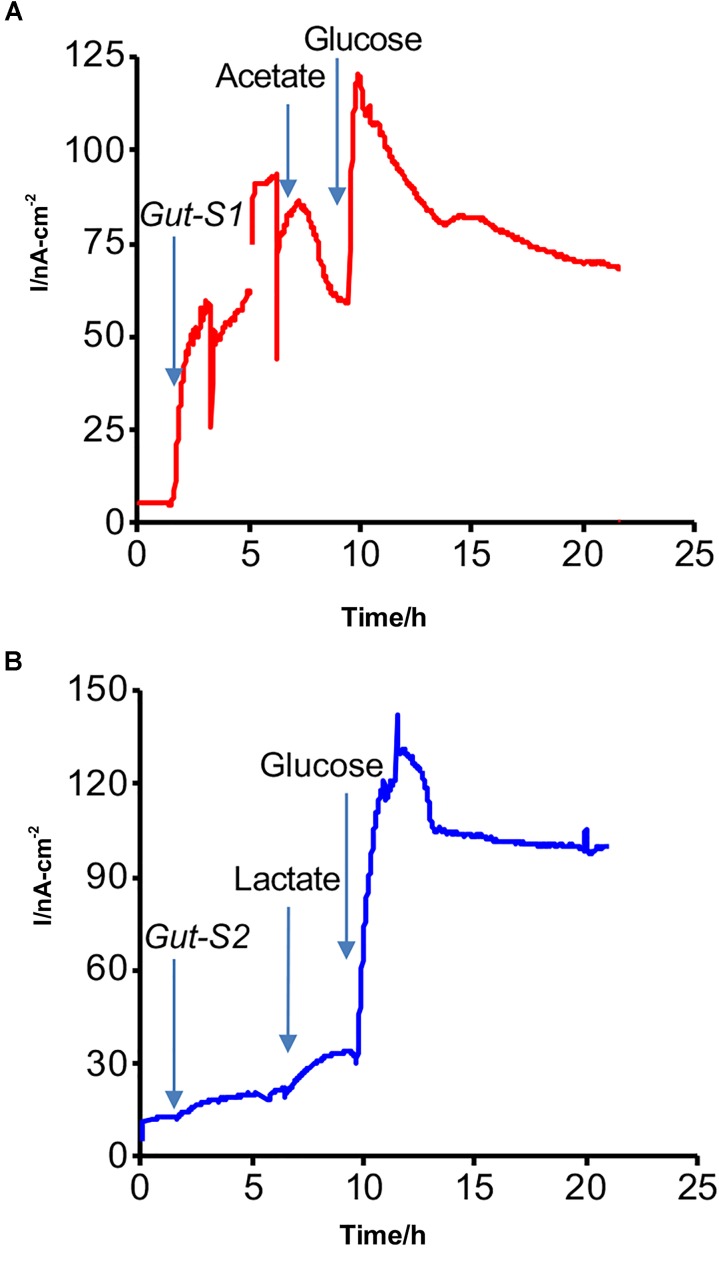
Representative current production versus time in isolated **(A)** *Gut-S1* and **(B)** *Gut-S2* measured with the ITO electrode poised at 0.4 V vs. SHE, initiated with the sterile DM1 medium containing only yeast extract as a carbon source at time = 0. At indicated times with arrows, microbes, acetate or lactate, and glucose were added.

An SA experiment with the isolated strains was performed using the DM1 medium without the addition of acetate or lactate at the start of the incubation (Figure 2). The anodic current was observed to increase once *Gut-S1* was added to the reactor with sterile media (Figure 2A), suggesting the microbial capability of EET for the anode in *Gut-S1*. However, this medium did not contain acetate, and hence, the current production was most likely due to the oxidation of the yeast extract, as no other organic source was present. To our surprise, upon addition of acetate (30 mM), the current production immediately decreased by 10% and continued to decrease gradually, followed by a short current recovery, suggesting that 30 mM acetate might damage *Gut-S1*, although we had been able to use acetate to enrich for the *Gut-S1* strain. The current increased to 120 nA cm^-2^ after the addition of glucose, demonstrating the microbial viability of *Gut-S1* and its ability to couple glucose oxidation with current production. These data strongly suggested that *Gut-S1* was enriched by the complex organic substrates of yeast extract, rather than by the acetate, implying that there was no microbial strain that could use the abundant acetate to outcompete the bacteria that could utilize the yeast extract.

In contrast, a gradual current increase of 15 nA cm^-2^ was observed upon lactate addition in the case of *Gut-S2* (Figure 2B), suggesting that lactate oxidation contributed to the current production. As glucose addition resulted in an immediate current increase in *Gut-S1* (Figure 2A) and *Gut-S2* (Figure 2B), the current production may be limited by the rate of the metabolic reaction in both strains. These results indicated that the lactate-oxidation metabolism takes the lead role for the enrichment of the *Gut-S2* cells on the electrode surface during the electrochemical enrichment.

**FIGURE 3:**
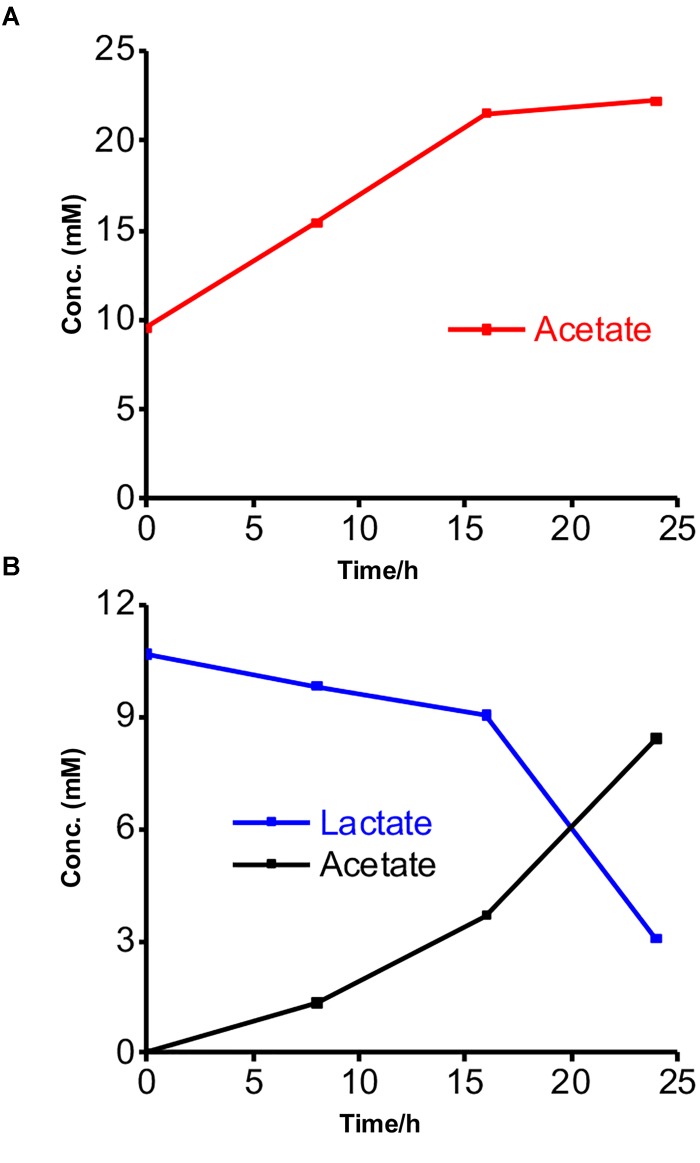
Metabolites concentrations measured at every 8-h time interval during the current production of isolated strains in the presence of acetate or lactate. **(A)** Acetate production in the presence of *Gut-S1* using DM2 containing 10 mM acetate and 0.5 g/L yeast extract. **(B)** Lactate consumption and acetate production in case of *Gut-S2* using DM2 containing 10 mM lactate and 0.5 g/L yeast extract. Similar tendency was observed in more than two individual experiments.

We further characterized the metabolism of the two strains and the EET mechanisms in these strains by electrochemistry and a metabolite assay in a different minimal medium DM2, which we usually use to characterize the current production capability and EET mechanism of *S. oneidensis* MR-1 (Okamoto et al., 2013; Saito et al., 2016; Rowe et al., 2017). This medium has a lower yeast extract concentration than DM1 and lacks trace minerals and vitamin solutions. Additionally, the acetate and lactate concentrations were reduced to 10 mM to eliminate the possibility of toxicity to the bacterial cells from the high organic concentrations. No significant change was observed in the current generation with and without acetate in the yeast extract-containing media for *Gut-S1*, signifying that EET was not coupled with acetate oxidation in the case of *Gut-S1* (Supplementary Figure S3). Furthermore, metabolite quantification for *Gut-S1* in IC showed that the acetate concentration increased and that it was not consumed, which was most likely due to the production of acetate from the oxidation of the yeast extract (Figure 3A). In the case of *Gut-S2*, we observed lactate oxidation and acetate production associated with current production (Figure 3B), indicating that lactate was not fermented but anaerobically respired. A slightly lower consumption of lactate, compared to the production of acetate, indicated the oxidation of the yeast extract to produce acetate in *Gut-S2*. These results demonstrated that *Gut-S2* has an EET capability associated with lactate oxidation, which is similar to the anaerobic respiration in the EET model microbial strain, *S. oneidensis* MR-1.

**FIGURE 4:**
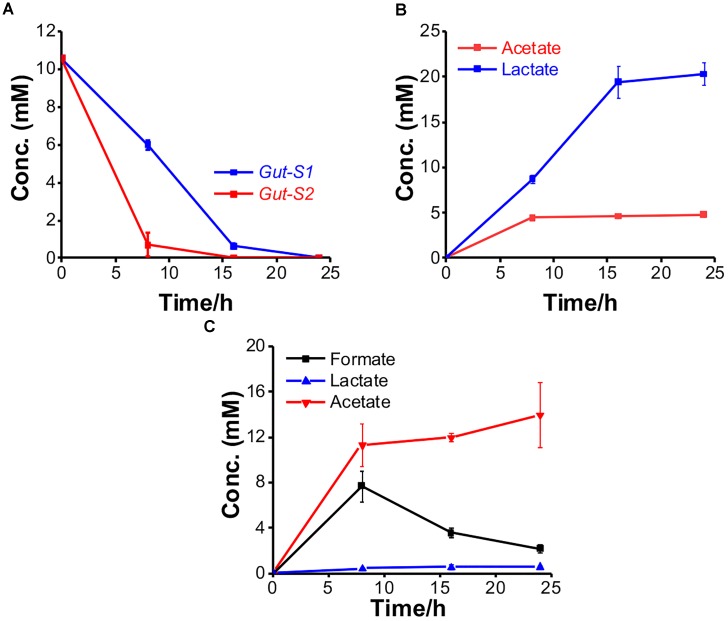
The metabolism of *Gut-S1* and *Gut-S2* during the current production associated with glucose fermentation. **(A)** Time course of glucose consumption and its metabolites concentration with **(B)** *Gut-S1* and **(C)** *Gut-S2* during current production using DM2 containing 10 mM glucose. The data shown are the mean values ± standard deviations of two replicate experiments.

To examine their ability to couple EET with fermentation, the metabolites were further explored using glucose in the electrochemical system. The consumption and production rates for glucose and the metabolites, respectively, were identified using *Gut-S1* and *Gut-S2* in DM2. Ten millimolars of glucose was completely consumed by both strains in 24 h, which is 50–80% faster than the consumption rate of lactate in *S. oneidensis* MR-1, indicating considerable microbial activity (Figure 4A). *Gut-S1* produced lactate and acetate (Figure 4B), while acetate and formate were the main metabolites for *Gut-S2* (Figure 4C). Lactate and formate are known as common end products for bacterial fermentation, and formate can be further oxidized to CO_2_ and H_2_ under anoxic conditions (Lim et al., 2014). The hydrogen produced in electrochemical cells may be oxidized at the ITO electrode surface and may contribute to the current generation by *Gut-S2*. A very low coulombic efficiency, i.e., less than 0.02%, was observed with both strains, based on the glucose consumption and coulombs generated. Given that this value is much lower than that of environmental bacteria, like *S. oneidensis* MR-1 (Bretschger et al., 2007), which was also observed in the similar electrochemical set up that was used in our current study (Okamoto et al., 2013), the role of EET in fermentation may be distinct from that of well-studied EET microbes, which has associated EET with anaerobic respiration.

### The EET Mechanism in the Two Isolates

**FIGURE 5:**
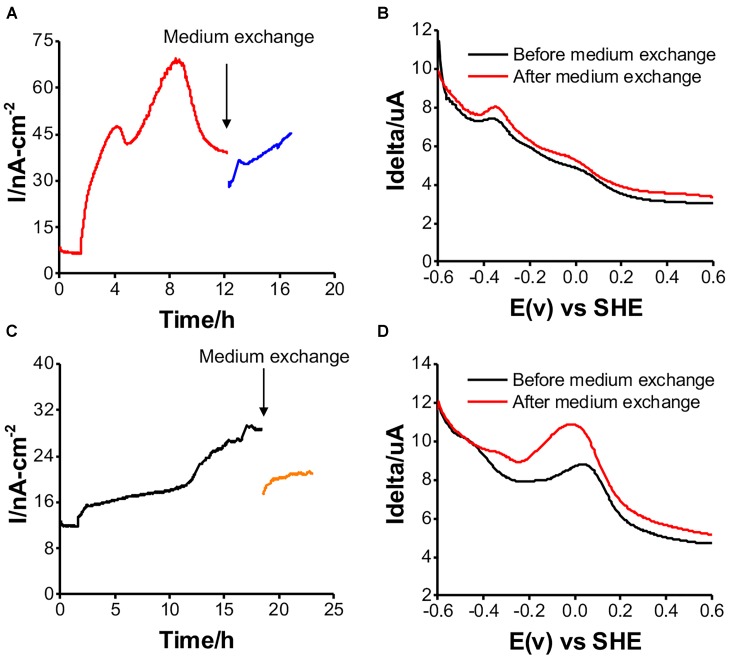
Medium exchange experiments for examining the contribution of soluble electron carrier to the current production, and DP voltammograms before and after medium exchange in reactors with *Gut-S1* (**A** and **B**) and *Gut-S2* (**C** and **D**), the similar tendency was observed in more than two experiments.

*Shewanella oneidensis* MR-1 has two potential EET mechanisms: direct electron transfer and indirect electron transport, mediated by a cell-surface enzyme and soluble electron carriers, respectively (Okamoto et al., 2013). To distinguish the two mechanisms for current production, medium exchange experiments have been performed to elucidate the contribution of the soluble electron carriers to the net current production (Marsili et al., 2008). After replacing the spent medium with fresh medium, there was a 20% decrease in the current for a short period of time. It recovered to the current level before the medium exchange in the case of *Gut-S1* (Figure 5A), suggesting a low contribution from the soluble electron carriers to the current production. Accordingly, the differential pulse (DP) voltammogram measured before and after the exchange of the electrolyte did not show a significant change, with the oxidative current peaks observed at around -0.35 and 0.0 V [vs. the standard hydrogen electrode (SHE)] (Figure 5B). In contrast, the current production decreased by approximately 50% and did not recover to the level from before the medium exchange for *Gut-S2* (Figure 5C); the oxidative peak potential also shifted significantly to the positive region in the DP voltammogram (Figure 5D), indicating a larger contribution from the soluble electron shuttle for the current production. Given that the *Gut-S2* cells most likely generate hydrogen, we observed the reduction in the formate concentration during current production (Figure 4C), which is a precursor for hydrogen generation. These data suggest that the *Gut-S2* current production could be assigned to the oxidation of fermentatively generated hydrogen. However, because a clear peak was still observed at -0.05 V (vs. SHE), even after the medium exchange, *Gut-S2* may also have a cell surface redox enzyme. Accordingly, for both *Gut-S1* and *Gut-S2*, cellular attachment on the electrode surface was confirmed by SEM analysis after the current production in the presence of glucose (Supplementary Figure S4). Taken together, the different peak positions and intensities of the oxidative peaks in the DP voltammograms suggested that different redox proteins are involved in these two strains.

## Discussion

In this study, the EET ability of isolates from a human gut sample was investigated, and the metabolic pathways were studied. Our data provide evidence that the physiological role of EET coupled with fermentation is somehow distinct from that of anaerobic microbial respiration. The magnitude of the anodic currents reported here, detected from these isolated strains, is significantly lower than those typically reported for the metal-reducing microbes usually investigated as EET model systems, and thus, these currents can be easily missed by traditional cultivation strategies. For example, *S. oneidensis* MR-1 produces two orders of magnitude more current than *Gut-S1* and *Gut-S2* (Xu et al., 2016). The low currents observed here also point to the lower ability of these isolated strains to gain cellular energy from external redox active surfaces. The physiological role of this mode of energy acquisition from external substrates should be further investigated in detail.

The phylogenetic analysis showed that *Gut-S1* and *Gut-S2* were similar to *E. avium* and *K. pneumoniae*, respectively. Both strains are facultative anaerobes, but not strict anaerobes, like the majority in the human gut. *E. avium* is a Gram-positive bacteria and is known as a rare human pathogen, and only a few case series exist (Lee et al., 2004). While the EET capability of this specific strain has not been reported, *K. pneumoniae* has been studied previously for its EET capability. This Gram-negative bacterium was isolated from subterranean forest sediment and investigated using glucose and starch as the carbon sources with successful current generation (Zhang et al., 2008). Furthermore, one study has reported an electron shuttling mechanism in *K. pneumoniae* based-microbial fuel cells (MFCs) (Deng et al., 2010). In our electrochemical analysis, these strains showed a spontaneous increase in current production with the fermentable carbon source such as glucose, and a less consumption of the nonfermentable carbon sources like acetate/lactate, indicating the fermentation associated EET.

In our study, one of the isolated strains was Gram-negative and the other was Gram-positive, and therefore, they should have different redox proteins for the transfer of the electrons to the electrode surface. Gram-negative bacteria are known to rely on cell surface-exposed cytochromes for the oxidation or reduction of extracellular minerals (Stams and Plugge, 2009; Wegener et al., 2015; White et al., 2016). Much less is known about the electroactivity of gram-positive bacteria than gram-negative ones. The cell envelope of gram-positive bacteria lacks an outer membrane, and the peptidoglycan layer is thicker (20–35 nm) (Beeby et al., 2013). Gram-positive bacteria are known to be poor in terms of their current production, but they can donate electrons to an external electrode (Pankratova and Gorton, 2017) and are frequent members of the microbial community in MFCs (Rabaey et al., 2004). A detailed understanding of this aspect may be important for devising strategies for pathogenicity control and the improvement of human health.

In addition to identifying new microbial candidates for EET, this study provides information on the role of EET associated with fermentation, distinct from that of anaerobic respiration. We believe that this new EET mode, coupled with fermentation, may open new windows for biotechnological applications and pathogenicity control models. It should be noted that while the focus here was on gut microbes, similar electrochemically active microorganisms with EET capability may be of interest in the vast range of other human pathogens and the external environment.

## Author Contributions

DN, MS, and AO designed and conducted the experiments. WM and AO wrote the manuscript.

## Conflict of Interest Statement

The authors declare that the research was conducted in the absence of any commercial or financial relationships that could be construed as a potential conflict of interest.
