# Supplementary material for: Isolation and Characterization of Human Gut Bacteria Capable of Extracellular Electron Transport by Electrochemical Techniques
Source: Front Microbiol. 2019 Jan 15;9:3267. doi: 10.3389/fmicb.2018.03267 (PMC6340925; doi:10.3389/fmicb.2018.03267)
Supplement: Supplementary file 1 [file Data_Sheet_1.docx]

**SUPPLIMENTARY INFORMATION**

**Isolation and Characterization of Human Gut Bacteria Capable of Extracellular Electron Transport by Electrochemical Techniques**

Divya Naradasu^1,2^†, Waheed Miran^1^†, Mitsuo Sakamoto^3,4^, Akihiro Okamoto^1,4,5^*

^1^International Center for Materials Nanoarchitectonics (WPI-MANA), National Institute for Materials Science (NIMS), 1-1 Namiki, Tsukuba, Ibaraki 305-0044, Japan

^2^Department of Advanced Interdisciplinary Studies, Rcast, Graduate School of Engineering, The University of Tokyo, 4-6-1 Komaba, Meguro-ku, Tokyo 153-8904 Japan

^3^Microbe Division/Japan Collection of Microorganisms, RIKEN BioResource Research Center, Tsukuba, Ibaraki 305-0074, Japan

^4^PRIME, Japan Agency for Medical Research and Development (AMED), Tsukuba, Ibaraki 305-0074, Japan

^5^Center for Sensor and Actuator Material, National Institute for Materials Science (NIMS), 1-1 Namiki, Tsukuba, Ibaraki 305-0044, Japan

*To whom all correspondence should be addressed.

E-mail: [okamoto.akihiro@nims.go.jp](mailto:okamoto.akihiro@nims.go.jp)

†Two authors equally contributed to the study.

**Figure S1.** Schematic illustration of enrichment procedures initiating from a human fecal sample. We electrochemically enriched a gut microbial consortium diluted to a concentration of 2 × 10^-9^ (v/v) poised at +0.2 V vs Ag/AgCl (sat. KCl), and isolated EET-capable bacteria by using 𝛿-MnO_2_-agar plate. The colonies forming the transparent spots in the dark brown agar plate were identified as those of EET-capable bacteria.

**Figure S2.** Electrochemical activity of gut microbes during enrichment phase a) first cycle and b) second cycle (the replacement of medium) with minimum medium. Reactor 1 and Reactor 2 were operated with acetate and lactate as electron donor, respectively.


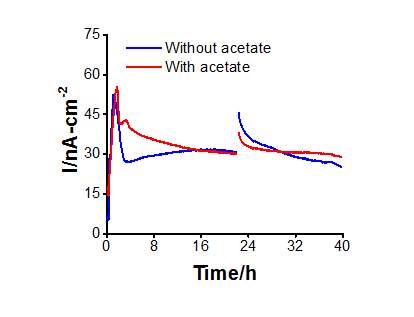


**Figure S3.** Representative current production data versus time in isolated *Gut-S1* with electrode poised at +0.2 V vs. Ag/AgCl (sat. KCl) initiated with (10 mM) and without acetate in DM2 medium.

**Figure S4.** Scanning electron microscope images of *Gut-S1* and *Gut-S2* biofilm attached on the electrode surface after 24 hours of current production with 10 mM glucose at +0.2 V vs. Ag/AgCl (sat. KCl).
